# Characterization of Plp, a phosphatidylcholine-specific phospholipase and hemolysin of *Vibrio anguillarum*

**DOI:** 10.1186/1471-2180-13-271

**Published:** 2013-11-27

**Authors:** Ling Li, Xiangyu Mou, David R Nelson

**Affiliations:** 1 Department of Cell and Molecular Biology, University of Rhode Island, 120 Flagg Rd., Kingston, RI 02881, USA; 2 College of Ocean and Earth Sciences, Xiamen University, Xiamen 361102, China; 3 Present address: Massachusetts General Hospital / Harvard Medical School, 65 Landsdowne St. PRB 425, Cambridge, MA 02139, USA

**Keywords:** *Vibrio anguillarum*, Vbriosis, Phospholipase, Hemolysis, Virulence

## Abstract

**Background:**

*Vibrio anguillarum* is the causative agent of vibriosis in fish. Several extracellular proteins secreted by *V. anguillarum* have been shown to contribute to virulence. While two hemolysin gene clusters, *vah1-plp* and *rtxACHBDE*, have been previously identified and described, the activities of the protein encoded by the *plp* gene were not known. Here we describe the biochemical activities of the *plp*-encoded protein and its role in pathogenesis.

**Results:**

The *plp* gene, one of the components in *vah1* cluster, encodes a 416-amino-acid protein (Plp), which has homology to lipolytic enzymes containing the catalytic site amino acid signature SGNH. Hemolytic activity of the *plp* mutant increased 2-3-fold on sheep blood agar indicating that *plp* represses *vah1*; however, hemolytic activity of the *plp* mutant decreased by 2-3-fold on fish blood agar suggesting that Plp has different effects against erythrocytes from different species. His_6_-tagged recombinant Plp protein (rPlp) was over-expressed in *E. coli*. Purified and re-folded active rPlp exhibited phospholipase A2 activity against phosphatidylcholine and no activity against phosphatidylserine, phosphatidylethanolamine, or sphingomyelin. Characterization of rPlp revealed broad optimal activities at pH 5–9 and at temperatures of 30-64°C. Divalent cations and metal chelators did not affect activity of rPlp. We also demonstrated that Plp was secreted using thin layer chromatography and immunoblot analysis. Additionally, rPlp had strong hemolytic activity towards rainbow trout erythrocytes, but not to sheep erythrocytes suggesting that rPlp is optimized for lysis of phosphatidylcholine-rich fish erythrocytes. Further, only the loss of the *plp* gene had a significant effect on hemolytic activity of culture supernatant on fish erythrocytes, while the loss of *rtxA* and/or *vah1* had little effect. However, *V. anguillarum* strains with mutations in *plp* or in *plp* and *vah1* exhibited no significant reduction in virulence compared to the wild type strain when used to infect rainbow trout.

**Conclusion:**

The *plp* gene of *V. anguillarum* encoding a phospholipase with A2 activity is specific for phosphatidylcholine and, therefore, able to lyse fish erythrocytes, but not sheep erythrocytes. Mutation of *plp* does not affect the virulence of *V. anguillarum* in rainbow trout.

## Background

*Vibrio anguillarum*, a highly motile marine member of the γ-Proteobacteria, is one of the causative agents of vibriosis, a fatal hemorrhagic septicemic disease of both wild and cultured fish, crustaceans, and bivalves [1]. Fish infected with *V. anguillarum* display skin discoloration and erythema around the mouth, fins, and vent. Necrotic lesions are also observed in the abdominal muscle [2]. Mortality rates in infected fish populations range as high as 30-100% [1,3]. Vibriosis has caused severe economic losses to aquaculture worldwide [1,3] and affects many farm-raised fish including Pacific salmon, Atlantic salmon, sea bass, cod, and eel [3,4]. *V. anguillarum* enters its fish host through the gastrointestinal tract (GI) and quickly colonizes this nutrient rich environment [2,5]. Garcia *et al*. [6] have shown that *V. anguillarum* grows extremely well in salmon intestinal mucus and that mucus-grown cells specifically express a number of different proteins, including several outer membrane proteins [6] and the extracellular metalloprotease EmpA [2,5].

Several genes have been reported to be correlated with virulence by *V. anguillarum*, including the *vah1* hemolysin cluster [7,8], the *rtx* hemolysin cluster [9], the siderophore mediated iron transport system [10], the *empA* metalloprotease [2,5], and the *flaA* gene [11]. Hemolytic activity of *V. anguillarum* has been considered to be the virulence factor responsible for hemorrhagic septicemia during infection [10]. We have identified two hemolysin gene clusters in *V. anguillarum* that contribute to the virulence of this pathogen [8,9]. One gene cluster, *rtxACHBDE*, encodes a MARTX toxin and its type I secretion system [9]. The second hemolysin gene cluster in *V. anguillarum* strain M93Sm contains the hemolysin gene *vah1* flanked by two putative lipase-related genes (*llpA* and *llpB*) immediately downstream and upstream by a divergently transcribed hemolysin-like gene (*plp*) that appears to function as a repressor of *vah1*-dependent hemolytic activity [8]. The *plp*-encoded protein has very high sequence similarity to phospholipases found in other pathogenic *Vibrio* species [8]. However, the enzymatic characteristics of Plp in *V. anguillarum* were not described.

Generally, phospholipases are divided into several subgroups depending on their specificity for hydrolysis of ester bonds at different locations in the phospholipid molecule. Phospholipases A (PLAs) cleave long chain fatty acids at sn-1 (PLA1) or sn-2 (PLA2) position from phospholipid to yield lysophospholipid and free fatty acid; phospholipases C (PLCs) cleave phospholipid into diacylglycerol and a phosphate-containing head group; and phospholipases D (PLDs) cleave phospholipid into phosphatidic acid and an alcohol. It is known that some phospholipid products are used as secondary messages, which play a central role in signal transduction [12].

In this study, we determined that *plp* encodes a phospholipase A2 in *V. anguillarum*, and then purified recombinant Plp protein (rPlp) from *E. coli* to investigate its biochemical properties. We also described the contribution and specificity of rPlp for hydrolysis of phospholipids, and its ability to lyse fish erythrocytes.

## Results

### Identification of a putative phospholipase gene plp

**Figure 1:**
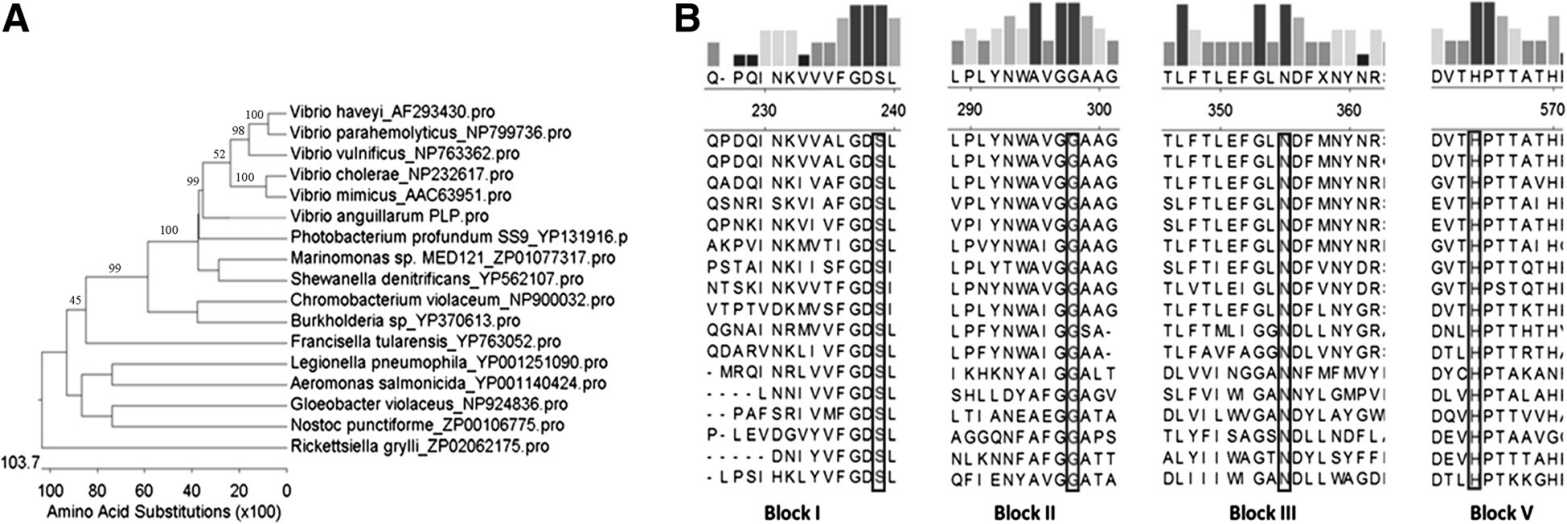
**The phylogenetic tree (A) and amino acid sequence alignment (B) of *V. anguillarum *Plp with members of the SGNH family.** The phylogenetic tree was analyzed by the Neighbor Joining (NJ) method with 1000 bootstraps, and node support values (as percentages) are labeled above the branch lines of the phylogenetic tree leading to the Plp homologues found in the genus *Vibrio*. Sequences of the 16 closest matches to Plp are aligned along the five conserved blocks of the SGNH family (Block IV not shown). The rectangle bars above the alignment indicate the amount of conservation of amino acid residues. The four residues conserved in all SGNH family members are boxed.

Previously, a putative phospholipase gene, *plp*, was identified in the *vah1* hemolysin cluster of *V. anguillarum* strain M93Sm [8]. The 1251-bp *plp* gene (Genbank accession EU650390) was predicted to encode a protein of 416 amino acids. A BLASTx [13] search revealed that the deduced Plp amino acid sequence exhibited homology with many lipolytic enzymes including the phospholipase/lecithinase/hemolysin of *Vibrio vulnificus* (identity, 69%; similarity, 82%); the lecithin-dependent hemolysin (LDH)/ thermolabile hemolysin (TLH) of *Vibrio parahaemolyticus* (identity, 64%; similarity, 80%); the lipolytic enzyme/hemolysin VHH of *Vibrio harveyi* (identity, 63%; similarity, 78%); and the thermolabile hemolysin of *Vibrio cholerae* (identity, 62%; similarity, 78%). The phylogenetic tree created by the Clustal-W program from 17 Plp homologous proteins revealed that while the most closely related Plp proteins were all from pathogenic members of the genus *Vibrio*, the Plp of *V. anguillarum* was an outlier among the *Vibrio* species, as demonstrated by the Neighbor Joining analysis (Figure 1). According to Flieger’s classification [14,15], the alignment of Plp with other homologous proteins indicated that Plp could be classified into subgroup B of this lipolytic family with its long N-terminal tail (data not shown) prior to the block I [14]. Additionally, close examination of the amino acid sequences of these enzymes revealed that the typical GDSL motif for lypolytic enzymes is not totally conserved in all of these 17 proteins, in which leucines (L) are replaced with isoleucines (I) in *Photobacterium*, *Marinomonas*, and *Shewanella* (Figure 1). In this case, *V. anguillarum* Plp should be considered as a member of the SGNH hydrolase family, based on the Molgaard’s suggestion that only four amino acids (S, G, N, and H) are completely conserved among the GDSL proteins [16].

### Plp affects hemolysis of fish erythrocytes

**Figure 2:**
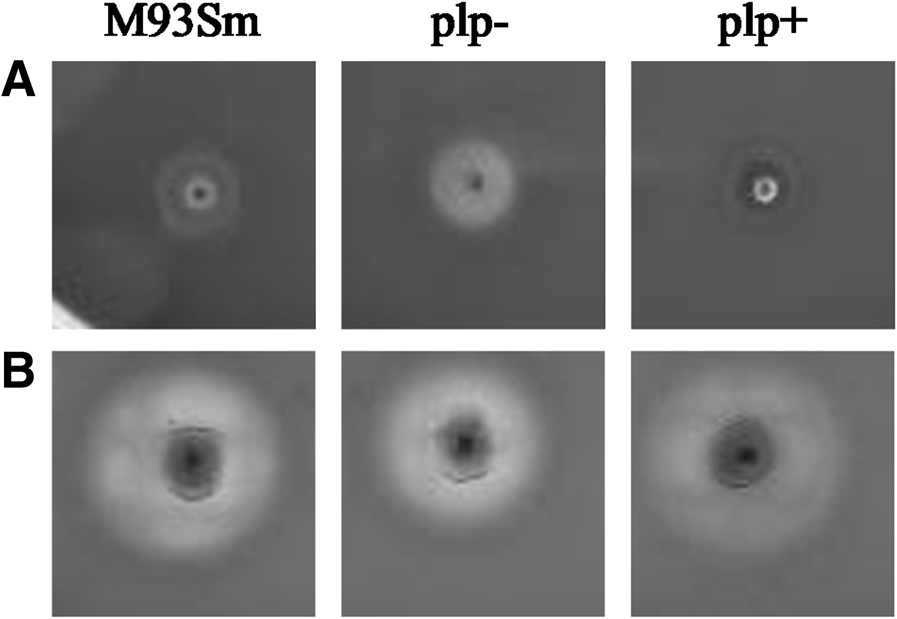
**Hemolytic activity of M93Sm and S262 (*plp*) on TSA-sheep blood agar (A) and LB20 + 5% rainbow trout blood agar (B).** A single colony of M93Sm and S262 was transferred onto each of the blood agars and incubated at 27°C for 24 h. The zones of hemolysis were measured and the diameters were given in the figure. This is a representative experiment from 3 replicate trials, each performed in triplicate.

The hemolysin gene *vah1* is divergently transcribed from *plp*[17]. Mutation of *plp* increased hemolytic activity by 2-3-fold on Trypticase soy agar plus 5% sheep blood (TSA-sheep blood) plate compared with wild type strain (M93Sm) (Figure 2A) [8]. Rock and Nelson [8] also demonstrated that the *plp* mutant had increased *vah1* transcription (by 2-4-fold), indicating that Plp is a putative repressor of *vah1*. Previously, we demonstrated that a double mutant in *vah1* and *rtxA* resulted in a hemolysis negative mutant when plated on TSA-sheep blood agar [9]. Similar results were observed when using Luria-Bertani broth plus 2% NaCl plus 5% sheep blood (LB20-sheep blood) agar (data not shown). However, on LB20 plus 5% rainbow trout blood (LB20-rainbow trout blood) agar, the *plp* mutant exhibited a smaller zone of hemolysis compared to wild type strain M93Sm (diameter: 9.5 ±0.5 mm vs. 12 ± 0.0 mm, *P* < 0.05) (Figure 2B); complementation of *plp* restored the hemolytic activity of the mutant strain (Figure 2B). Similar results were observed when using LB20 plus 5% Atlantic salmon blood agar (data not shown), suggesting that the ability of Plp to lyse erythrocytes is dependent upon the source of erythrocytes and, therefore, their lipid composition.

### Plp has phospholipase A2 activity

**Figure 3:**
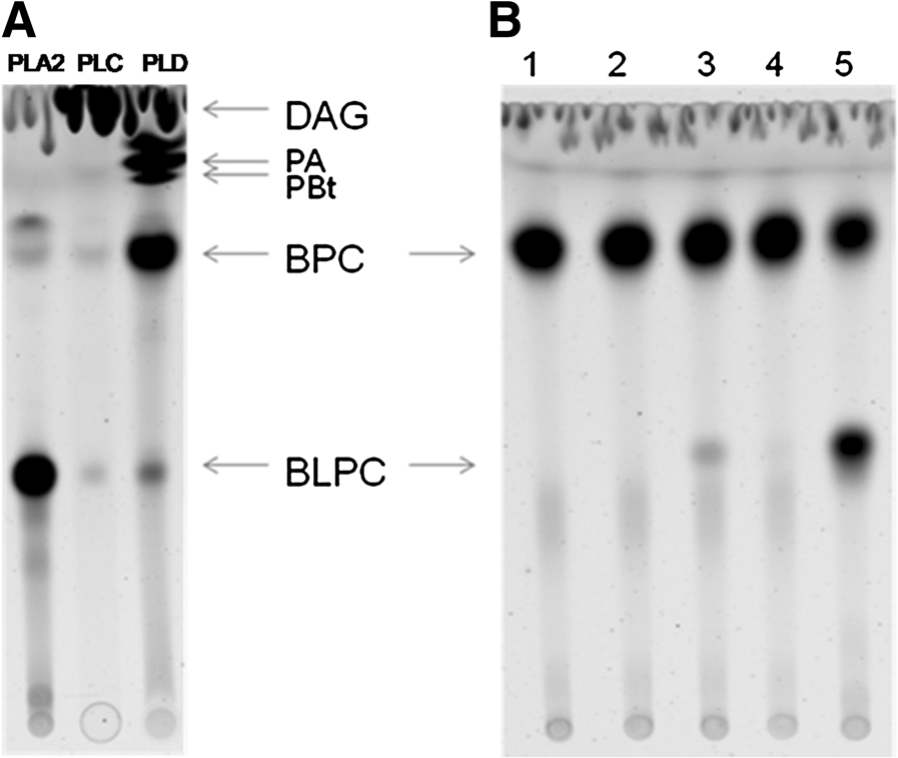
**Thin-layer chromatography (TLC) demonstrates phospholipase A2 activity of Plp.** BODIPY-labeled phosphatidylcholine (BPC) was incubated with various standard enzymes or sample preparations for 1 h at 37°C. Subsequently, the lipids were extracted and separated by TLC. **(A)** The cleavage patterns of BPC by standard proteins PLA2, PLC, and PLD were able to distinguish the different phospholipase activities. **(B)** Cleavage patterns of BPC by supernatants (lanes 2 and 3) and cell lysates (lanes 4 and 5) from *E. coli* DH5α containing cloned *plp* (lanes 3 and 5) or just the cloning vector pSUP202 (lanes 2 and 4). Lane 1 contains only BPC incubated in the presence of PBS buffer. BLPC, BODIPY-labeled lysophosphatidylcholine; PA, phosphatic alcohol; PBt, phosphaticbutanol; DAG, di-acylglycerol.

Thin layer chromatography (TLC) was used to examine the pattern of phospholipid cleavage by Plp. BODIPY-labeled phosphatidylcholine (BPC) was incubated with various enzyme standards, including phospholipase A2 (PLA2), phospholipase C (PLC), or phospholipase D (PLD). TLC analysis revealed distinct cleavage patterns (Figure 3A) by these standard enzymes indicating that BPC was an appropriate substrate to examine Plp activity. Cell lysate prepared from *E. coli* strain S299, which contains the shuttle plasmid pSUP202-*plp* that was able to complement the *plp* mutation in *V. anguillarum*[8], cleaved BPC to yield BODIPY-lysophosphatidylcholine (BLPC) (Figure 3B, lane 5) plus unlabeled free fatty acid (FFA) that is not detectable. The cleavage products were identical to those generated by PLA2 (Figure 3B) and demonstrate that Plp has phospholipase A2 activity. Additionally, the culture supernatant from S299 had only ~5% of the activity of that in cell lysate, indicating that Plp accumulated in the cell lysate instead of being secreted by the *E. coli* strain. No phospholipase activity was detected in PBS buffer or in *E. coli* DH5α containing only the pSUP202 vector (Figure 3B). Further, phospholipase A2 activity was examined in various subcellular fractions prepared from *E. coli* strain S299, including cytoplasm, cytoplasmic membrane, and outer membrane fractions. Most Plp activity was detected in Tween-20 soluble membrane fraction, indicating that Plp was mainly localized in the cytoplasmic membrane of *E. coli* S299 (data not shown). No BODIPY-labeled free fatty acid (FFA) (at sn-1 position) was detected in the TLC analysis when an apolar solvent was used (data not shown), and BODIPY-labeled LPC was not further degraded by Plp in the reaction, indicating that Plp had no lysophospholipase or phospholipase B activity.

### Enzymatic characteristics of rPlp protein

To examine the enzymatic characteristics of Plp, the entire coding sequence of *plp* was cloned and inserted into the expression vector pQE60, which adds a His_6_ (His-6×) tag to the carboxyl end of Plp. The over-expressed recombinant Plp (rPlp) formed inclusion bodies in *E. coli*. To recover active rPlp, purification of the inclusion bodies followed by solubilization under mild conditions and re-folding was performed as described in the Methods. Purity of refolded rPlp protein was confirmed by SDS-PAGE and silver staining (data not shown). The final concentration of purified rPlp protein was 8 μg/ml with a recovery of <10%.

**Figure 4:**
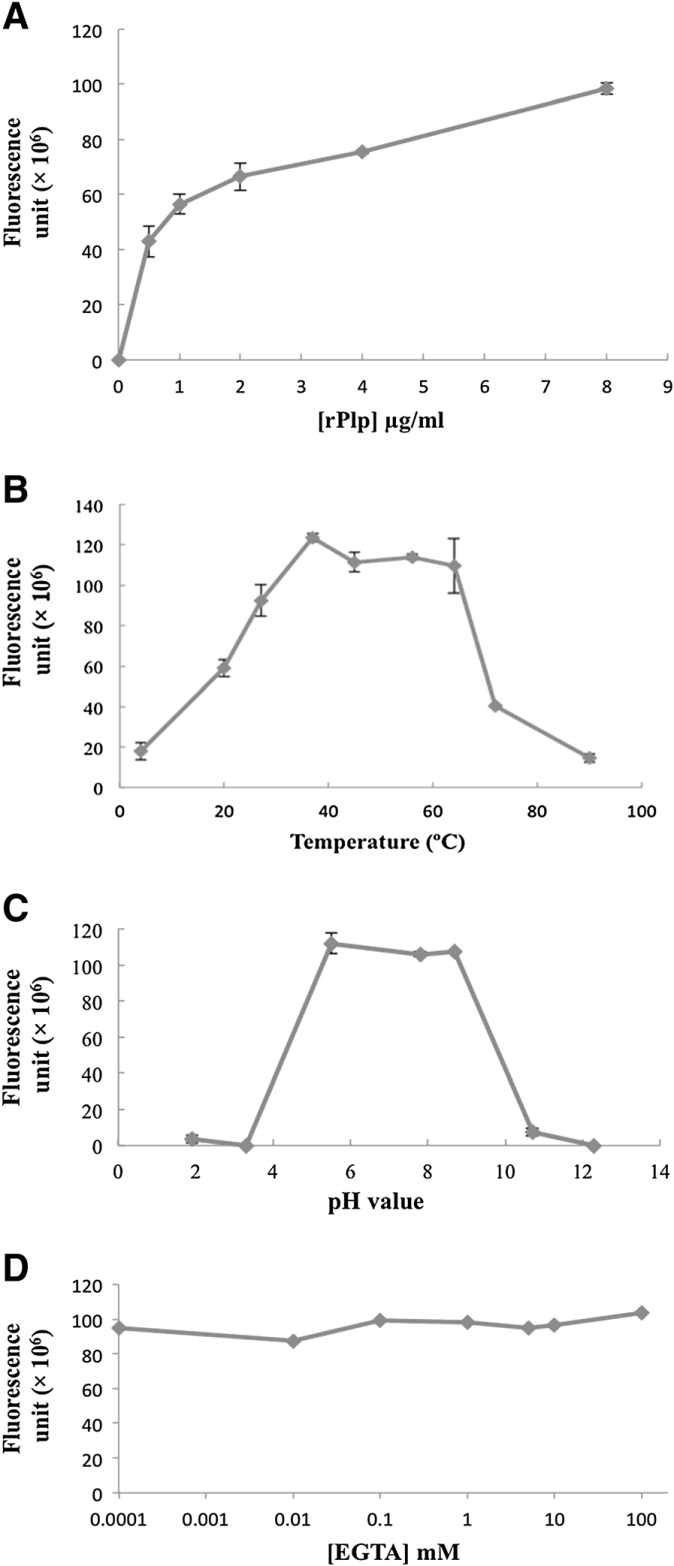
**Effects of chemical and physical conditions on rPlp activity. (A)** Effect of rPlp concentration on enzymatic activity. **(B)** The effect of temperature on rPlp activity. **(C)** The effect of pH on rPlp activity. **(D)** The effect of EGTA rPlp activity.

Subsequently, the enzymatic characteristics of refolded rPlp were examined under various chemical and physical conditions. The enzymatic activity of rPlp positively correlated to its concentration from 1 μg/ml to 8 μg/ml (Figure 4A); therefore, 4 μg/ml rPlp protein was routinely used in other activity assays. The enzymatic activity unit of refolded rPlp (1 unit = amount of protein that cleaves 1 μmole of BODIPY-PC per minute) was about 2,500-fold higher than standard PLA2 enzyme extracted from porcine pancreas, which indicated that Plp had a high activity against the BPC phospholipid substrate. Plp enzyme activity exhibited a broad temperature optimum from 37°C to 64°C (Figure 4B) with 75% activity retained at 27°C and 50% activity at 20°C. While rPlp activity rapidly decreased at temperatures above 70°C, the enzyme retained full activity at 64°C for at least 1 h. The data demonstrate that rPlp is a relatively themostable phospholipase.

The effect of pH on enzyme activity was determined for pH values ranging from 2 to 12. The data showed that rPlp had a broad pH optimum from pH 5.3 to pH 8.7 with activity dropping off rapidly at pH values above and below the optimum (Figure 4C). rPlp activity was not affected by treatment with the chelating reagents EGTA (Figure 4D) or EDTA (data not shown) at concentrations up to 100 mM. Additionally, treatment with divalent metal ions, such as calcium or magnesium had no effect on activity (data not shown).

### Plp is a secreted protein in *V. anguillarum*

**Figure 5:**
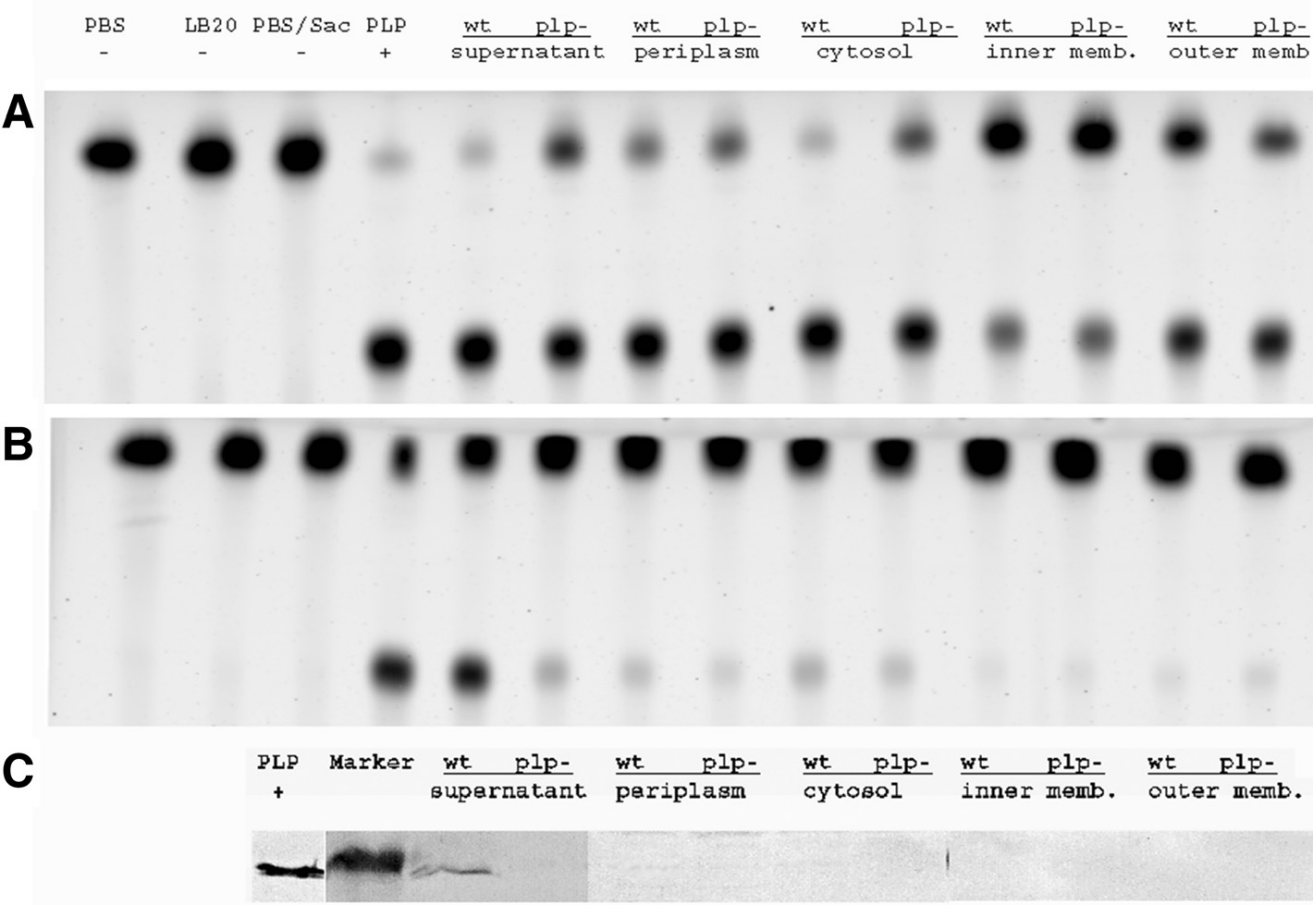
**The phospholipase activity assays detected by TLC of various cell fractions prepared from wild type (wt) strain M93sm and *plp *mutant strain S262 (plp-) were performed at 37°C (A) and 64°C (B).** PBS buffer, LB20, and PBS buffer + 1% sarcosylate were served as negative controls. The refolded rPlp protein (PLP +) served as positive control. The top spots on each chromatogram are the BPC substrate and the bottom spots are the BLPC reaction product. The proteins from the same cell fractionation preparations were analyzed by SDS-PAGE and Western blot analysis **(C)** as described in the Methods. The refolded rPlp protein was served as positive control.

Subcellular fractions from *V. anguillarum* strains M93Sm and S262 (*plp*) were prepared and phospholipase A2 activity examined using BPC and TLC. Initial studies revealed that at 37°C phospholipase A2 activity was detected in all cell fractions, including the culture supernatant, periplasm, cytoplasm, cytoplasmic membrane, and outer membrane, from both M93Sm and S262 (Figure 5A). However, when the assay was performed at 64°C (to inactivate heat labile phospholipases), phospholipase A2 activity in S262 was significantly decreased in all fractions including the supernatant (Figure 5B). Additionally, when the assay was performed at 64°C for M93Sm subcellular fractions, only the culture supernatant exhibited phospholipase activity against BPC (about 100-fold higher activity compared to the phospholipase activity of the S262 supernatant). The data demonstrated that Plp was secreted into the culture supernatant of *V. anguillarum,* which corresponds with *in silico* analysis of the deduced Plp amino acid sequence (Accession number DQ008059) by SignalP that Plp has a signal peptide [18]. TLC results also revealed that there was at least one other protein in *V. anguillarum* M93Sm exhibiting phospholipase A2 activity besides the secreted, heat stable Plp protein. This was a themolabile PLA2 activity inactivated at 64°C.

In order to confirm that Plp was localized in the supernatant of *V. anguillarum*, protein samples prepared from various subcellular fractions were separated by SDS-PAGE and analyzed by western blot analysis using polyclonal rabbit anti-Plp antiserum. An immuno-reactive band of ~45 kDa was detected only in the supernatant of M93Sm, but was absent in the supernatant of *plp* mutant (Figure 5C). Taken together with the phospholipase A2 activity data, these data indicate that Plp is a secreted protein in *V. anguillarum*.

### rPlp has a specific activity against phosphatidylcholine

**Figure 6:**
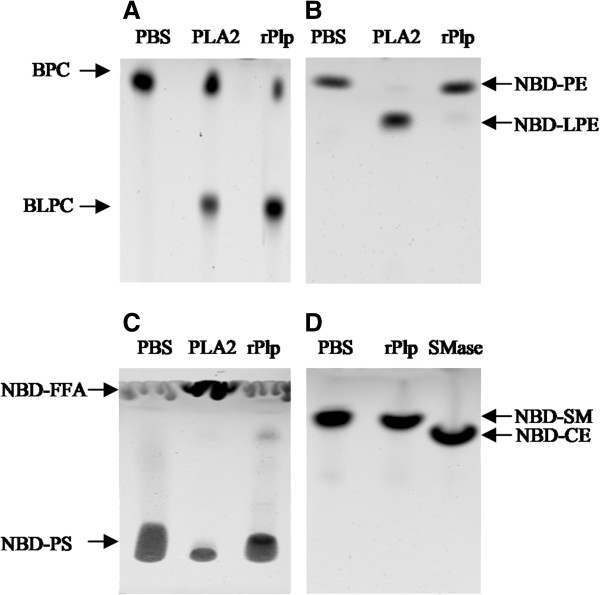
**rPlp activity determined by TLC analysis.** BPC **(A)**, NBD-PE **(B)**, NBD-PS **(C)**, and NBD-Sm **(D)** were used as phospholipid substrates to examine the specificity of rPlp. Phosphate-buffered saline (PBS) was used as a negative control, and PLA2 enzyme from porcine pancreas as a positive control. BPC: BODIPY-labeled phosphatidylcholine; BLPC: BODIPY-labeled lysophosphatidylcholine; NBD-PE: NBD-labeled phosphatidylethanolamine; NBD-LPE: NBD-labeled lysophosphatidylethanolamine; NBD-PS: NBD-labeled phosphatidylserine; NBD-FFA: NBD-labeled free fatty acid; NBD-SM: NBD-labeled sphingomyelin; NBD-CE: NBD-labeled ceramide.

Various fluorescently-labeled phospholipid substrates (described in Methods) were used to determine the specificity of the rPlp protein. rPlp exhibited high activity against phosphatidylcholine, cleaving BPC to yield BLPC and free fatty acid (Figures 3 and 6A). However, rPlp had almost no activity against both NBD-phosphatidylethanolamine (NBD-PE) (Figure 6B) and NBD-phosphatidylserine (NBD-PS) (Figure 6C), showing only 2% and 5%, respectively, of the activity of the standard PLA2 protein against each of the substrates. The data indicated that the rPlp protein does not efficiently cleave either phosphatidylethanolamine or phosphatidylserine. Additionally, unlike the standard sphingomyelinase, rPlp was not able to cleave the NBD-sphingomyelin into the NBD-ceramide and phosphocholine (Figure 6D), indicating that rPlp had no sphingomyelinase activity. Taken together, the data demonstrated that Plp is a phosphatidylcholine-specific PLA2 enzyme.

### rPlp is able to lyse the fish erythrocytes directly

**Figure 7:**
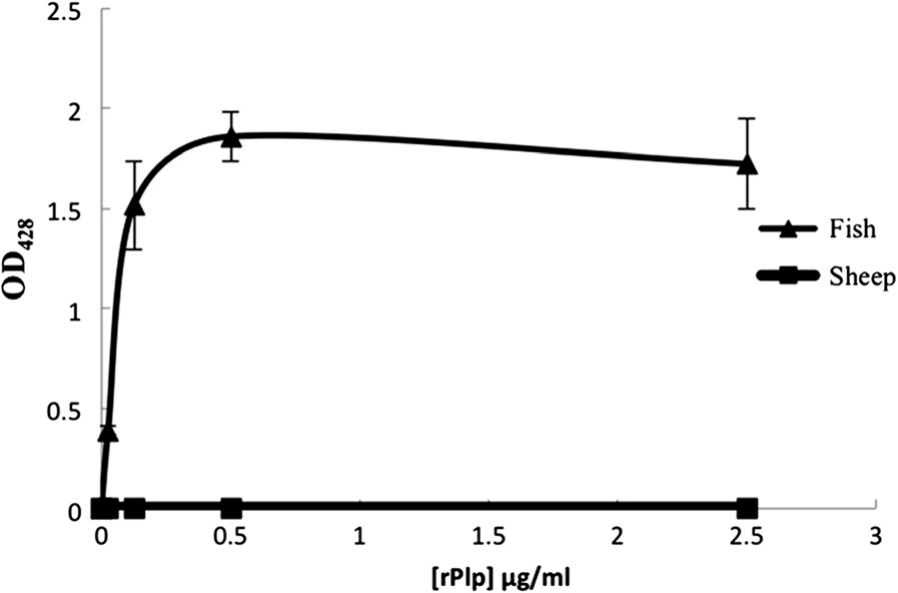
**Lysis of Atlantic salmon erythrocytes by recombinant Plp protein (rPlp).** 500 μl 5% fish (triangle) and sheep (square) erythrocytes were incubated with various concentration rPlp at 27°C for 20 h. The lysis of erythrocytes was measured at 428 nm. Erythrocyte resuspension buffer (10 mM Tris–HCl, 0.9% NaCl, pH 7.2) was used as negative control. All values were calculated from three independent experiments. Error bars show one standard deviation.

Membrane phospholipid compositions are quite varied among the animal species, especially for phosphatidylcholine. It is known that phosphatidylcholine makes up 58% of the total phospholipid in fish erythrocytes [19]; however, no phosphatidylcholine is found in sheep erythrocytes [20]. In order to determine whether the differential hemolysis observed for *plp* mutants of *V. anguillarum* (Figure 2) is due to the activity of Plp against PC, we tested the ability of purified rPlp to lyse Atlantic salmon erythrocytes. Addition of recombinant Plp resulted in the lysis of Atlantic salmon erythrocytes, with the amount of lysis directly related to the amount of rPlp added to the blood suspension (Figure 7). In contrast, addition of rPlp to a suspension of sheep erythrocytes resulted in no lysis of those cells (Figure 7). These data show that Plp has phosphatidylcholine-specific phospholipase A2 activity and can directly lyse fish erythrocytes.

### Plp is one of the hemolysins of *V. anguillarum*

Previously, we demonstrated that there are two major hemolysin gene clusters in the M93Sm, the *vah1* cluster [8] and the *rtxA* cluster [9]. Mutation of both *vah1* and *rtxA* completely eliminated the hemolytic activity of M93Sm on TSA-sheep blood agar [9]. Mutation of the *plp* gene resulted in 2-3-fold increased hemolytic activity on TSA-sheep blood agar because *vah1* expression increased both transcriptionally and translationally in the *plp* mutant, indicating that Plp is a putative repressor of *vah1*[9]. Plp also has hemolytic activity against fish erythrocytes due to its phosphatidylcholine-specific activity (Figures 6 and 7). To investigate the relationship of the three hemolysins, culture supernatants obtained from various *V. anguillarum* strains (Table 1) were used to examine the hemolytic activity against the fish blood (Table 2).

**Table 1:** Bacterial strains and plasmids used in this study

**Strain or plasmid**	**Description**	**Reference**
*V. anguillarum* strains		
M93sm	Spontaneous Sm^r^ mutant of M93 (serotype O2a); parental strain isolated from a diseased ayu (*Plecoglossus altivelis*) from Lake Biwa, Japan	[2]
JR1	Sm^r^ Cm^r^*vah1*; insertional *vah1* mutant of M93Sm	[8]
XM21	Sm^r^ Cm^r^ Tc^r^*vah1+*; *vah1* complement strain of JR1	This study
S262	Sm^r^ Cm^r^*plp*; insertional *plp* mutant of M93Sm	This study
XM31	Sm^r^ Cm^r^ Tc^r^*plp+*; *plp* complement strain of S262	This study
S123	Sm^r^ Cm^r^*rtxA*; insertional *rtxA* mutant of M93Sm	[9]
JR3	Sm^r^ Cm^r^ Km^r^*vah1 plp*; insertional *vah1*mutant of JL01	[8]
S183	Sm^r^ Cm^r^ Km^r^*vah1 rtxA*; insertional *rtxA* mutant of S171	[9]
XM62	Sm^r^ Cm^r^ Km^r^ Tc^r^*vah1+ rtxA*; *vah1* complement strain of S183	This study
S187	Sm^r^ Cm^r^ Km^r^*plp rtxA*; insertional *rtxA* mutant of JL01	This study
XM90	Sm^r^ Cm^r^ Km^r^*vah1 plp rtxA*; insertional *plp* mutant of S264	This study
XM93	Sm^r^ Cm^r^ Km^r^ Tc^r^*vah1 plp + rtxA*; *plp* complement strain of XM90	This study
JL01	Sm^r^ Km^r^*plp*; mini-Tn10Km insertion into *plp*	[8]
S171	Sm^r^ Km^r^*vah1*; allelic exchange *vah1* mutant	[9]
S264	Sm^r^ Km^r^*vah1 rtxA*; allelic exchange *vah1* and *rtxA* mutant	This study
*E. coli* strains		
Sm10	*thi thr leu tonA lacY supE recA* RP4-2-Tc::Mu::Km (λ *pir*)	[21]
S253	Km^r^ Cm^r^; Sm10 containing plasmid pNQ705-*plp*	This study
S118	Km^r^ Cm^r^; Sm10 containing plasmid pNQ705-*rtxA*	[9]
S250	Km^r^ Cm^r^; Sm10 containing plasmid pDM4-*rtxA5'*	This study
S252	Km^r^ Cm^r^; Sm10 containing plasmid pDM4-*rtxA5'-rtxA3'*	This study
U21	Km^r^ Ap^r^ Tc^r^; Sm10 containing plasmid pSUP202-*vah1*	This study
U31	Km^r^ Ap^r^ Tc^r^; Sm10 containing plasmid pSUP202-*plp*	This study
M15	Nal^S^ Str^S^ Rif^S^*thi*^–^*lac*^–^*ara*^+^*gal*^+^*mtl*^–^ F^–^*recA*^+^*uvr*^+^*lon*^+^ (pREP4)	QIAGEN, USA
S238	Km^r^ Ap^r^; M15 containing plasmid pQE30UA-plp	This study
S269	Km^r^ Ap^r^; M15 containing plasmid pQE60-*plp*	This study
Plasmid		
PCR2.1	Km^r^ Ap^r^; Cloning vector	Invitrogen, USA
pNQ705-1	Cm^r^; suicide vector with R6K origin	[22]
pNQ705-vah1	Cm^r^; for insertional *vah1*mutation	[8]
pNQ705-*plp*	Cm^r^; for insertional *plp* mutation	This study
pNQ705-*rtxA*	Cm^r^; for insertional *rtxA* mutation	[9]
pDM4	Cm^r^ SacBC^r^; suicide vector with R6K origin	[11]
pDM4-*rtxA5'-rtxA3'*	Cm^r^ SacBC^r^; for allelic exchange *rtxA* mutation	This study
pSUP202	Cm^r^ Ap^r^ Tc^r^; *E. coli* – *V. anguillarum* shuttle vector	[21]
pSUP202-*vah1*	Ap^r^ Tc^r^; for complementation of *vah1*	This study
pSUP202-*plp*	Ap^r^ Tc^r^; for complementation of *plp*	This study
pQE-30 UA	Ap^r^; expression vector with N-terminal His_6_-tag	QIAGEN, USA
pQE30UA-*plp*	Ap^r^; for expression of rPlp that is used to make anti-Plp	This study
pQE60	Ap^r^; expression vector with C-terminal His_6_-tag	QIAGEN, USA
pQE-60-*plp*	Ap^r^; for expression of rPlp for enzymatic activity analysis	This study

**Table 2:** **Hemolytic activity of culture supernatant from *V. anguillarum *** **wild-type and various *V. anguillarum *** **mutant strains against rainbow trout blood cells**

***V. anguillarum *** **strain or treatment**	**Hemolytic activity (Relative to wild-type control ± SD)**^**a**^
M93Sm	1.00 (±0.12)
JR1 (*vah1*)	0.98 (±0.16)
XM21 (*vah1+*)	1.20 (±0.28)
S262 (*plp*)	0.28 (±0.09)^b^
XM31 (*plp+*)	0.99 (±0.04)
S123 (*rtxA*)	0.94 (±0.22)
JR03 (*plp vah1*)	0.14 (±0.09)^b^
S183 (*vah1 rtxA*)	1.51 (±0.29)
XM62 (*vah1+ rtxA*)	0.73 (±0.03)
S187 (*plp rtxA*)	0.12 (±0.09)^b^
XM90 (*vah1 rtxA plp*)	−0.04 (±0.09)^b^
XM93 (*vah1 rtxA plp+*)	1.33 (±0.01)
Water (positive control)	1.15 (±0.16)

^a^Hemolytic activity assays carried out using the tube assay method as described in the Methods. Hemolysis by M93Sm was given the value of 1.00. The data are representative of two independent experiments, each with three replicates, ± one standard deviation (SD).

^b^Statistically different from hemolytic activity for M93Sm (*P* < 0.05).

In contrast to the strong hemolytic activity against 5% rainbow trout blood mixed with culture supernatant from the wild type strain M93Sm, hemolytic activity of culture supernatant from strain S262 (*plp*) declined by >70% (Table 2). Additionally, all mutants containing a knockout of *plp* exhibited significant declines (*P* < 0.05) in hemolytic activity. The triple hemolysin mutant, XM90 (*plp vah1 rtxA*) had no ability to lyse fish erythrocytes (Table 2). However, mutations in either *vah1* or *rtxA*, but not *plp*, resulted in little or no decline in hemolytic activity against fish erythrocytes compared to supernatants from wild type cells (Table 2). Further, complementation of *plp* restored the hemolytic activity of supernatants from both the *plp*-complemented strains (XM31, *plp +* and XM93, *vah1 rtxA plp*+) (Table 2). Taken together, these data clearly demonstrate that Plp is the major hemolytic enzyme responsible for the lysis of rainbow trout erythrocytes by culture supernatants of *V. anguillarum*.

### Plp is not a major virulence factor for *V. anguillarum* during fish infection

**Figure 8:**
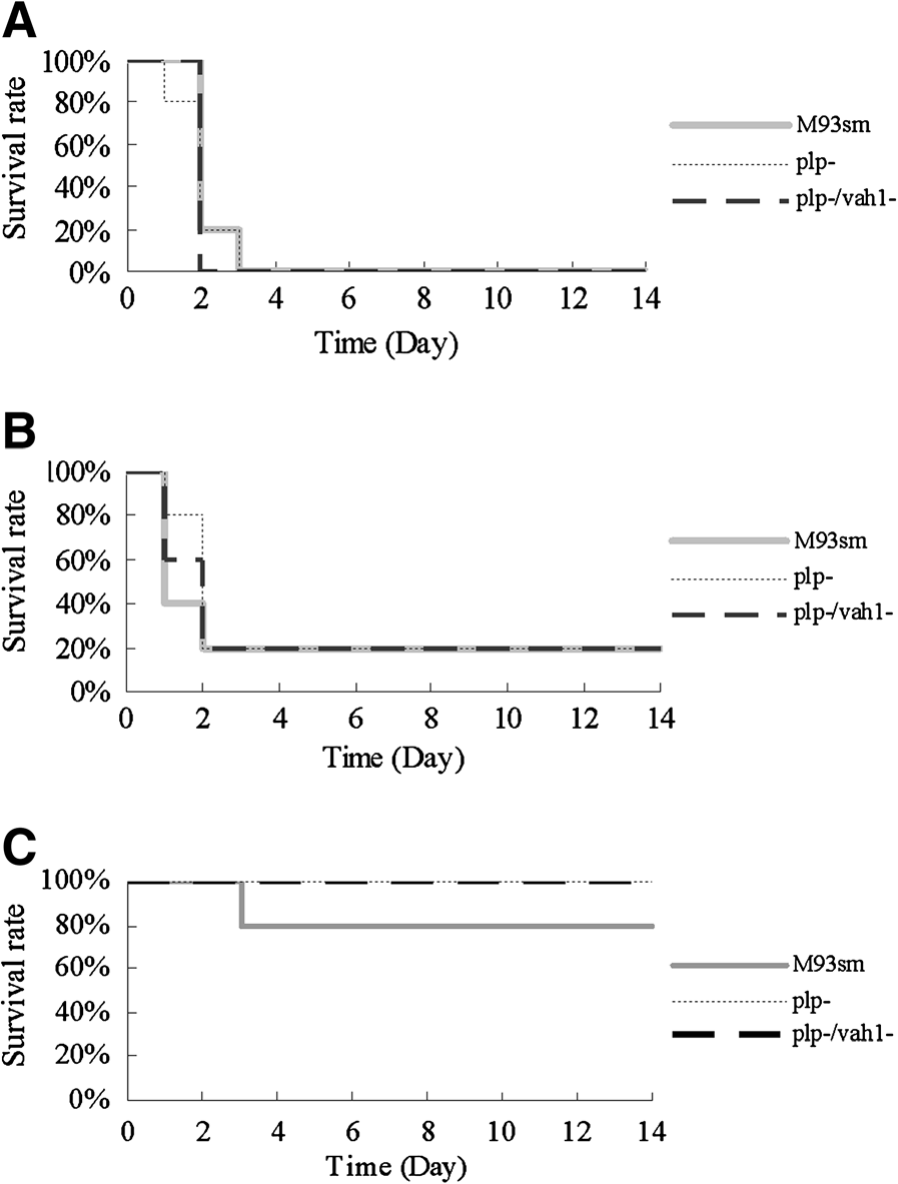
**Survival rate of rainbow trout injected IP with wild type (M93Sm, solid grey line) and mutant (*plp*, grey dotted line; *plp vah1*, black dashed line) strains of *V. anguillarum *strains at doses of A) 3 × 10**^**6**^**, B) 3 × 10**^**5 **^**or C) 3 × 10**^**4**^ **CFU/fish.** No statistically significant difference was observed between the strains.

In order to determine whether the *plp* gene affects virulence in fish, an infection study was performed by inoculating rainbow trout by IP injection with either the wild type strain M93Sm or mutant strains S262 (*plp*) or JR03 (*vah1 plp*). The results of this experiment (Figure 8) indicated that there were no statistical differences in mortality between the three strains. This suggested that mutation of either *plp* or *vah1* or both genes did not decrease the virulence of M93Sm. These results are consistent with our previous observations that *rtxA* is a major virulence factor of M93sm and that mutation of *vah1* does not affect virulence [8], and demonstrate that Plp is not a major virulence factor in the *V. anguillarum* M93Sm.

## Discussion

In this report, we describe the characteristics of the *V. anguillarum* phospholipase protein (Plp) encoded by *plp*, and its contribution to the hemolytic activity of *V. anguillarum*. Specifically, we show that Plp is a secreted phospholipase with A2 activity with specificity for phosphatidylcholine. The enzyme has a broad temperature optimum (37 – 64°C) and a broad pH optimum (pH 5.5 – 8.7). Phospholipases are broadly distributed among the *Vibrionaceae* and often contribute to the virulence of the pathogenic members of this family. For example, the TLH or LDH of *V. parahaemolyticus*[23-25] was the first well-studied lecithin-dependent PLA/lysophospholipase [26]. A lecithinase (encoded by *lec*) was also identified in *V. cholerae*[27]. Fiore *et al.*[27] found that a *lec* mutant strain was unable to degrade lecithin and the culture supernatant exhibited decreased cytotoxicity. However, the mutant did not exhibit decreased fluid accumulation in a rabbit ileal loop assay, suggesting that fluid accumulation in animals is not affected by lecithinase activity. Additionally, the phospholipase A (PhlA) in *V. mimicus* was found to exhibit hemolytic activity against trout and tilapia erythrocytes and was cytotoxic to the fish cell line CHSE-214 [28]. Recently, the *V. harveyi* hemolysin (VHH) was shown to be a virulence factor during flounder infection and also had phospholipase activity on egg yolk agar [29]. Rock and Nelson [8] reported that the putative phospholipase gene (*plp*) from *V. anguillarum* exhibits 69% amino acid identity with the *V. cholerae lec* gene. Both *plp* and *lec* are located divergently adjacent to a hemolysin gene (*vah1* and *hlyA*, respectively) [8,27]. Additionally, Rock and Nelson [8] demonstrated that functional *plp* repressed transcription of its adjacent hemolysin gene, *vah1*, in *V. anguillarum*. However, the enzymatic characteristics of Plp in *V. anguillarum* were not described.

Usually, phospholipases are divided into phospholipases A (A1 and A2), C, and D according to the cleavage position on target phospholipids. Most of lipolytic enzymes contain a putative lipid catalytic motif (GDSL) that was previously demonstrated in other bacterial and eukaryotic phospholipases [30]. However, Molgaard [16] demonstrated that four amino acid residues (SGNH) form a catalytic site, and are conserved in all members of the phospholipase family; therefore, phospholipases were re-named as the SGNH subgroup of the GDSL family [30]. Multiple alignment analysis of 17 phospholipase homologues (Figure 1) demonstrates that *V. anguillarum* Plp belongs to the SGNH hydrolase subgroup, since the GSDL motif was not fully conserved in these proteins (Figure 1). Recently, it was reported that mutation of the serine residue in the SGNH motif resulted in the complete loss of the phospholipase and hemolytic activities of VHH in *V. harveyi*[31] demonstrating the importance of this motif on the activity of phospholipase.

In contrast to the similarities of their catalytic motifs, the biochemical characteristics of bacterial phospholipases appear to be variable. For example, *V. mimicus* PhlA has a phospholipase A activity, which cleaves the fatty acid at either sn-1or sn-2 position, but no lysophospholipase activity [28]. Two phospholipases identified from mesophilic *Aeromonas sp.* serogroup O:34 show phospholipase A1 and C activity [32]. In addition, TLH of *V. parahaemolyticus* has PLA2 and lysophospholipase activity*,* and demonstrates a loss of activity at 55°C for 10 min [23]. In this report, we show that *V. anguillarum* Plp has PLA2 activity, and is able to maintain activity at 64°C for 1 h (Figures 6 and 7). Therefore, the enzymatic characteristics of specific phospholipases are distinct even when they all belong to the SGNH hydrolase family (Figure 1).

Phospholipases have been implicated in the pathogenic activities of a number of bacteria [33,34]. It is known that phospholipase activities often lead to cell destruction by degrading the phospholipids of cell membranes [33,35]. However, the relationships between phospholipases and virulence are not always clear. While the purified rPlp exhibits strong hemolytic activity against Atlantic salmon erythrocytes (Figure 7), Rock and Nelson [8] showed that a knock-out mutation of either the *plp* gene or the *vah1* gene in *V. anguillarum* did not affect virulence of *V. anguillarum* during an infection study on juvenile Atlantic salmon. In this report, we show that when groups of rainbow trout are infected with either a *plp* mutant or a *plp vah1* double mutant there is no significant difference in mortalities compared to fish infected with the wild type strain. Our data suggest that neither *plp* nor *vah1* are major virulence factors during pathogenesis of salmonids. It was also reported that the deletion of lecithinase (Lec) activity in *V. cholerae* did not significantly diminish fluid accumulation in the rabbit ileal loop assay, indicating the lecithinase activity does not contribute significantly to enterotoxin activity [27]. Lec is a homologue of Plp [8]. In contrast, the direct IP injection of purified *V. harveyi* VHH protein caused the death of flounder with an LD_50_ of about 18.4 μg protein/fish [29]. The rPhlA of *V. mimicus* also has a direct cytotoxic effect on the fish cell line CHSE-214 [28] suggesting that this phospholipase is a virulence factor during fish infection. In addition, the lecithinase purified from *A. hydrophila* (serogroup O:34) has been shown to be an important virulence factor to rainbow trout and mouse [32]. We note that infection experiments in both Atlantic salmon and rainbow trout demonstrate that mutation of *plp* does not attenuate virulence. We propose that *V. anguillarum* is able to compensate for the loss of Plp-mediated hemolytic activity *in vivo* by up-regulating the transcription of *vah1*, as previously described by Rock and Nelson [8]. Additionally, transcription of *rtxA* is also increased in a *plp* mutant (Mou and Nelson, unpublished data).

Generally, the hemolytic activity of phospholipases is dependent upon the hydrolysis of the phospholipids that reside in the erythrocyte membrane. Erythrocytes contain various phospholipids including phosphatidylcholine (PC), phosphatidylethanolamine (PE), phosphatidylserine (PS), phosphatidylinositol (PI), and sphingomyelin (SM). PC makes up 58% of the total erythrocyte phospholipids in the Atlantic salmon [36], but only 34% and 1% in rabbit and sheep erythrocytes, respectively [20]. Taken together with the high specificity of rPlp for PC (Figure 6), it was not surprising that rPlp was able to lyse the fish erythrocytes, but not sheep erythrocytes (Figure 7), and that the *plp* mutant had decreased hemolytic activity on LB20-fish blood agar (Figure 2). Our results are consistent were those reported for *V. mimicus* PhlA [28] and *V. harveyi* VHH [29], in which PhlA and VVH specifically lyse the fish erythrocytes.

We have previously reported that there are two hemolysin gene clusters in *V. anguillarum* M93Sm, the *vah1-plp* cluster and *rtxACHBDE* cluster [9] and have described their regulation by H-NS and HlyU [17,37]. Mutation of both *vah1* and *rtxA* results in the loss of all hemolytic activity on TSA-sheep blood agar [9], which is consistent with the data reported here that Plp has no activity on sheep erythrocytes. We have also previously demonstrated that Plp is a putative repressor of Vah1, since mutation of *plp* increases *vah1* expression by 2–3 fold [8]. In this report, we examined the hemolytic activity of various hemolysin mutants using freshly collected Rainbow trout blood (Table 2) to investigate the relationships among three hemolysins of *V. anguillarum*. While culture supernatants from two of the three single mutants (JR1 and S123) and one of three double mutants (S183) exhibited ≥94% of the hemolytic activity as supernatants from the wild type strain M93Sm (Table 2), the hemolytic activity of one single mutant (S262, *plp*) and two double mutants (JR03, *plp vah1* and S187, *plp rtxA*) were reduced to 28%, 14%, and 12% of that in M93Sm, respectively. Our data indicate that only the loss of the *plp* gene has a significant effect on hemolysis of fish erythrocytes by *V. anguillarum* culture supernatant, while the loss of *rtxA* and/or *vah1* has little effect. Further, supernatant from the hemolysin triple mutant XM90 (*vah1 rtxA plp*) exhibits no hemolytic activity on fish blood compared to M93Sm (Table 2), indicating that Vah1, RtxA, and Plp are responsible for all secreted hemolytic activity by *V. anguillarum*. Finally, complementation of any *plp* mutant with *plp* (*in trans*) restores hemolytic activity to *V. anguillarum* culture supernatant (Table 2).

## Conclusion

*V. anguillarum* Plp is a secreted hemolysin with phosphatidylcholine-specific phospholipase A2 activity. The ability of Plp to digest the abundant phosphatidylcholine found in the membrane of fish erythrocytes causes their lysis. The three hemolysins, Plp, Vah1 and RtxA, account for all hemolytic activity in *V. anguillarum* culture supernatant under the experiment conditions described in this study. Finally, infection studies in rainbow trout demonstrate that the *plp* and *vah1* genes are not required for virulence.

## Methods

### Bacterial strains, plasmids, and growth conditions

All bacterial strains and plasmids used in this report are listed in Table 1. *V. anguillarum* strains were routinely grown in Luria-Bertani broth plus 2% NaCl (LB20) [38], supplemented with the appropriate antibiotic, in a shaking water bath at 27°C. *E. coli* strains were routinely grown in Luria-Bertani broth plus 1% NaCl (LB10). Antibiotics were used at the following concentrations: streptomycin, 200 μg/ml; ampicillin, 100 μg/ml (Ap^100^); chloramphenicol, 20 μg/ml (Cm^20^) for *E. coli* and 5 μg/ml (Cm^5^) for *V. anguillarum*; kanamycin, 50 μg/ml (Km^50^) for *E. coli* and 80 μg/ml (Km^80^) for *V. anguillarum*; tetracycline, 15 μg/ml (Tc^15^) for *E. coli,* 1 μg/ml (Tc^1^) for *V. anguillarum* grown in liquid medium*,* and 2 μg/ml (Tc^2^) for *V. anguillarum* grown on agar plates.

### Insertional mutagenesis

Insertional mutations were made by using a modification of the procedure described by Milton *et al.*[28]. Briefly, primers (Table 3) were designed based on the target gene sequence of M93Sm. Then a 200–300 bp DNA fragment of the target gene was PCR amplified and ligated into the suicide vector pNQ705-1 (GenBank accession no. KC795685) after digestion with *Sac*I and *Xba*I. The ligation mixture was introduced into *E. coli* Sm10 by electroporation using BioRad Gene Pulser II (BioRad, Hercules, CA). Transformants were selected on LB10 Cm^20^ agar plates. The construction of the recombinant pNQ705 was confirmed by both PCR amplification and restriction analysis. The mobilizable suicide vector was transferred from *E. coli* Sm10 into *V. anguillarum* by conjugation. Transconjugants were selected by utilizing the chloramphenicol resistance gene located on the suicide plasmid. The incorporation of the recombinant pNQ705 was confirmed by PCR amplification.

**Table 3:** Primers used in this study

**Primers**	**Sequence (5' to 3', italicized sequences are designed restriction sites)**	**Purpose and description**	**Reference**
Pm262	ATCGA*GGATCC*ATGAAACTAATGACGTTATTG	For whole Plp protein, forward	This study
Pm263	ATCGA*AGATCT*TTGAAATTGAAATGACGCGAG	For whole Plp protein, reverse	This study
Pm212	GACACCTCACAATATGAAATAAAA	For truncated Plp protein, forward	This study
Pm213	TTTGAGCTGCGGGGCTTTGGTTGC	For truncated Plp protein, reverse	This study
Pm261	ATCGA*GAGCTC*GCAGAATCGTGACTGACGCCG	For insertional *plp* mutation, forward, with *Sac*I site	This study
SD Lip/Heme R1	GCTAG*TCTAGA*ACGGATACCACCTCAGA	For insertional *plp* mutation, reverse, with XbaI site	[8]
pr1	GGG*GAATTC*TTATTCAAATTGAAATGACGCGAG	For *plp* complement, forward, with EcoRI site	This study
pr2	GGG*ACCGGT*GAATACCCATTTTTTATTTTTTC	For *plp* complement, reverse, with AgeI site	This study
pr3	GTT*GAATTC*GTATTTTCTGCAATCGCCATG	For *vah1* complement, forward, with EcoRI site	This study
pr4	GGG*ACCGGT*CTATTTTATAATAAATTGAATACCAT	For *vah1* complement, reverse, with AgeI site	This study
Pm256	ATCGA*CTCGAG*CTGGAGAAGATGTACTCTGCG	For allelic exchange *rtxA* mutation, flanking the 5' region, forward, with XhoI site	This study
Pm257	ATCGA*TCTAGA*CGTATCATCTACAGCTTTTGC	For allelic exchange *rtxA* mutation, flanking the 5' region, reverse, with XbaI site	This study
Pm258	ATCGA*TCTAGA*TTATATTAATCATGTCTTTTATGGG	For allelic exchange *rtxA* mutation, flanking the 3' region, forward, with XbaI site	This study
Pm259	ATCGA*GAGCTC*CTGATTGCCTAGCAGTAGCCC	For allelic exchange *rtxA* mutation, flanking the 3' region, reverse, with SacI site	This study
pr7	CAGGAAACAGCTATGACCATGATTACG	For sequencing of the DNA fragment inserted in pCR2.1 TA-ligation site	This study
pr8	CTACGGGCTTGAGCGTGACAATC	For sequencing of the DNA fragment inserted in pSUP202 AgeI site	This study
pr25ex	GCTGTCCCTCCTGTTCAGCTACTGACGGGGTGGTGCG	For sequencing of the DNA fragment inserted in pNQ705-1 Multi-cloning site	This study

### Allelic exchange mutagenesis

The allelic exchange *rtxA* mutation in *V. anguillarum* S264 was made by using a modification of the procedure described by Milton *et al.*[28]. The 5′ region of *rtxA* was amplified using the primer pair pm256 and pm257 (Table 3), digested with XhoI and XbaI, and then cloned into the region between the XhoI and XbaI sites on pDM4 (GenBank accession no. KC795686), deriving pDM4-*rtxA5′*. The 3′ region of *rtxA* was amplified using the primer pair pm258 and pm259 (Table 3), digested with XbaI and SacI, and then cloned into the region between the XbaI and SacI sites on the pDM4-*rtxA5′*. The resulting pDM4-*rtxA5′-rtxA3′* was transformed into *E. coli* Sm10 to produce the transformant strain S252, which was mated with *V. anguillarum* S171 (*vah1*). Single-crossover transconjugants were selected with LB20 Kan^80^ Sm^200^ Cm^5^ plates and, subsequently, double-crossover transconjugants (resulting in a 3345 bp deletion in C-terminal of RtxA) were selected with LB20 Kan^80^ Sm^200^ 5% sucrose plates. The resulting *V. anguillarum* colonies were transferred to TSA-sheep blood agar (Northeast Laboratories Service, Waterville, ME) and screened for none-hemolytic colonies (*vah1 rtxA*). The resulting colonies were checked for the desired allelic exchange using PCR amplification.

### Complementation of mutants

The various mutants were complemented by cloning the appropriate target gene fragment into the shuttle vector pSUP202 (GenBank accession no. AY428809) as described previously by [8]. Briefly, primers (Table 3) were designed with EcoRI and AgeI sites and then used to amplify the entire target gene plus ~500 bp of the 5′ and ~200 bp 3′flanking regions from genomic DNA of *V. anguillarum* M93Sm. The DNA fragment was then ligated into pSUP202 after digestion with *EcoR*I and *Age*I, and the ligation mixture was introduced into *E. coli* Sm10 by electroporation using a BioRad Gene Pulser II. Transformants were selected on LB10 Tc^15^ Ap^100^ agar plates. The complementing plasmid was transferred from *E. coli* Sm10 into the *V. anguillarum* mutant by conjugation. Transconjugants were selected by tetracycline resistance (Tc^2^). The transconjugants were then confirmed by PCR amplification and restriction digestion.

### Bacterial conjugation

Bacterial conjugation were carried out using the procedure modified from Varina *et al.*[39]. Briefly, 100 μl *V. anguillarum* grown overnight was added into 2.5 ml nine salts solution (NSS) [40]; 100 μl *E. coli* culture overnight was added into 2.5 ml 10 mM MgSO_4_. The resulting *V. anguillarum* and *E. coli* suspension was mixed, vacuum filtered onto an autoclaved 0.22-μm-pore-diameter nylon membrane (Millipore, USA), placed on an LB15 agar plate (LB-plus-1.5% NaCl), and allowed to incubate overnight at 27°C. Following incubation, the cells were removed from the filter by vigorous vortex mixing in 1 ml NSS. Cell suspensions (70 μl) were spread on LB20 plated with appropriate antibiotics and the plates were incubated at 27°C until *V. anguillarum* colonies were observed (usually 24 to 48 h).

### Cloning, over-expression, purification, and refolding of the Plp protein

The whole length of the *plp* gene (stop codon not included) was amplified by PCR with a sense primer introducing a BamHI site and an antisense primer introducing BglII site, respectively. Genomic DNA extracted from *V. anguillarum* M93Sm was used as template. The amplified PCR product was digested with BamHI and BglII, and ligated into a pQE60 (QIAGEN, USA) vector, which was also cut with BamHI and BglII. The ligation mix was transformed into *E. coli* M15 (pREP4) and clones with pQE60-plp were selected on LB10 agar containing kanamycin and ampicillin. A clone harboring plasmid pQE60-plp was selected and the plasmid DNA sequence isolated from the clone confirmed by sequencing. The clone was designated as S269. Subsequently, *E. coli* strain S269 was grown at 37°C in 500 ml LB10 broth to OD_600_ = 0.5, and isopropyl-β-D-thiogalactopyranoside (IPTG) was added to the culture (final concentration, 1 mM) to induce the expression of Plp. Then, the induced *E. coli* cells grown for 4 h at 37°C were harvested at 8000 × *g* for 10 min. The cell pellet was stored at −20°C overnight to improve lysis. Inclusion bodies of Plp were crudely purified using Cellytic B reagent (Sigma, USA). Refolding of Plp protein from the inclusion body preparation was carried out using a modification of the method described by Santa *et al.*[41]. Briefly, 500 μl of purified inclusion body (2 mg protein/ml) was completely solubilized in 1 ml of 50 mM Tris buffer (pH 12) containing 2 M urea. The solubilized Plp was diluted into 20 ml dilution buffer (50 mM Tris–HCl, pH 8.0; 0.2 M glycine; 10% glycerol; 2 M urea; 0.5 mM EDTA, and 0.2 mM DTT) at 4°C. No aggregation was observed during the dilution. The diluted Plp protein was dialyzed with the addition of 500 ml 50 mM Tris–HCl (pH8.0) until the total dialysis volume up to 3 L. The dialyzed Plp protein was concentrated with QIAGEN Ni-NTA Protein Purification Kit (QIAGEN) under native purification condition according to the instructions of the manufacturer. The protein concentration was determined using the BCA protein assay (Pierce).

### Hemolytic assays

The hemolytic activity of *V. anguillarum* strains was measured by two methods. First, single *V. anguillarum* colonies were transferred onto TSA-sheep blood agar, LB20-sheep blood agar (LB20 agar plus 5% sheep blood with heparin, obtained from Hemostat Laboratories) or LB20-fish blood agar (LB20 agar plus 5% rainbow trout or Atlantic salmon blood with heparin). Hemolytic activity of each colony was determined by measuring hemolytic zone surrounding the colonies after 24 h at 27°C. Additionally, the level of hemolytic activity was also quantitated using a microcentrifuge tube assay. The tubes contained 500 μl 5% erythrocytes (fish or sheep, suspended in 10 mM Tris-Cl, pH 7.5 – 0.9% NaCl buffer) were mixed with 500 μl of bacterial supernatant or rPlp and incubated for 20 h at 27°C. The samples were centrifuged at 1500 × g for 2 min at 4°C, and the optical density of the resulting supernatant was read at 428 nm.

### Phospholipase assay and thin-layer chromatography (TLC) analysis

Phospholipase assays were performed *in vitro* with a BODIPY-phosphatidylcholine (BPC or 2-decanoyl-1-(*O*-(11-(4,4-difluoro-5,7-dimethyl-4-bora-3a,4a-diaza-*s*-indacene-3-propionyl)amino)undecyl)-*sn*-glycero-3-phosphocholine; Invitrogen), NBD-phosphatidylethanolamine (NBD-PE, *N*-(NBD-Aminododecanoyl)L-1,2-dihexanoyl-*sn*-glycero-3-phosphoethanolamine; Sigma), NBD-phosphatidylserine (NBD-PS or 1-Palmitoyl-2-[12-[(7-nitro-2-1,3-benzoxadiazol-4-yl)amino]dodecanoyl]-*sn*-Glycero-3-Phospho-L-Serine; Avanti Polar Lipid), NBD-sphingomyelin (NBD-SM, N-[12-[(7-nitro-2-1,3-benzoxadiazol-4-yl)amino]dodecanoyl]-Sphingosine-1-Phosphocholine; Avanti Polar Lipid). 20 μM phospholipid substrates (10 μl) were reacted with an equal volume (10 μl) of various samples, and incubated at different conditions, as described for each experiment. For some experiments, purified standard phospholipases were used: PLA2 (Sigma) from porcine pancreas, PLC (Sigma) from *Clostridium perfringens*, and PLD (Sigma) from cabbage. The reaction products were analyzed by thin-layer chromatography (TLC). Briefly, 20 μl of 1-butanol was added to the above reaction mixes (20 μl), followed by vigorous vortex mixing for 30 s and centrifugation (10,000 × g, 1 min). The upper lipid extract layer (5 μl) was loaded onto a plastic-backed silica gel G60 plate without fluorescent indicator (Sigma) and air-dried for 20 min. TLC was performed either with chloroform-methanol–water-acetic acid (45/45/10/1 by vol.) when BODIPY-PC was used as the substrate, or with chloroform-methanol-acetic acid (60/20/1 by vol.) when NBD-PE, NBD-PS, or NBD-SM used as the substrates. For some experiments, an apolar solvent (n-hexane (70): diethyl ether (30): acetic aid (4)) was used. Fluorescence was detected and quantified using a Typhoon 9410 laser scanner.

### Subcellular fractionation

*V. anguillarum* cells were fractionated as described previously [6] and the subcellular location of Plp determined. Briefly, 100 ml NSS-washed overnight grown bacterial cells were resuspended in 10 ml of ultrapure water for 20 min to cause osmotic shock and centrifuged (10,000 × *g*, 5°C, 10 min) to collect the periplasmic fraction (the supernatant). The remaining pellets were washed twice with ultrapure water and lysed by sonication (four cycles at 35% power for 20 s, then allowed to cool for 1 min). The sonicated cells were centrifuged (10,000 × g, 5°C, 20 min) to remove cell debris and any unlysed cells, and the supernatant cell lysate was separated by ultracentrifugation (200,000 × *g*, 1 h, 4°C) to yield the cytosolic (supernatant) and membrane (pellet) fractions. The membrane fraction was treated with 1% Sarkosyl to obtain Sarkosyl-soluble (inner membrane) and -insoluble (outer membrane) fractions. Protein concentration in various fractions was measured using BCA protein determination kit (Pierce).

### Preparation of polyclonal antibody

Truncated Plp protein was over-expressed and purified to serve as the antigen to create polyclonal antibody against Plp. Briefly, primer Pm212 and Pm213 (listed in Table 3) were used to amplify central portion of the *plp* gene, which encodes the truncated Plp protein (amino acid 93 to 293). PCR product was ligated into pQE30UA vector (QIAGEN), and transformed into *E. coli* M15 and transformants were selected on LB10 agar containing kanamycin and ampicillin. Plasmid DNA was purified and the sequence confirmed by DNA sequencing. Protein purification was performed under denaturing conditions according to the instructions of the manufacturer (QIAGEN, USA) and protein purity was determined by SDS-PAGE and Coomassie blue staining. Subsequently, the purified truncated Plp was used as antigen to prepare polyclonal antibody in two New Zealand White rabbits (Charles River Lab, MA). Briefly, 1 ml purified antigen (concentration = 100 μg/ml) was vigorously mixed with 1 ml TiterMax Gold adjuvant (Sigma) into a homogeneous suspension. About 10 ml of blood was withdrawn from the rabbits before immunization as a control. For the first injection, antigen-adjuvant mix was subcutaneously injected at 4 sites (over each shoulder and thigh; 100 μl/site). The rabbits were boosted with single injections of antigen-adjuvant (100 μl) at day 28, 42, and 56. Blood was withdrawn 7–10 days after the 2^nd^ and 3^rd^ boosts to test the titer of antiserum using the western blot analysis. Antiserum with a high titer (> 1: 10,000) was aliquoted and stored at −70°C.

### Sodium dodecyl sulfate-polyacrylamide gel electrophoresis (SDS-PAGE) and Western blot analysis

Purified proteins or other protein samples were separated in 10% SDS-polyacrylamide gels. Prestained protein standards (Bio-Rad) and Laemmli sample buffer (Sigma) were used in all gels. Electrophoresis was performed at 100 V for 60–90 min. Gels were stained with either Coomassie blue G-250 or silver stain (Pierce, USA) to visualize the protein bands. Alternatively, proteins were transferred to nitrocellulose membranes for western blot analysis using the mini-Protean II system (Bio-Rad). Protein transfers were performed as described by Towbin *et al.*[42] at 100 V for 1 h. Nitrocellulose membranes were blocked with the addition of 5% skim milk. Detection of specific protein bands was accomplished by reacting the blot with the 1:5000 diluted anti-Plp antibody, followed by the addition of the secondary antibody goat anti-rabbit IgG conjugated with peroxidase, and then developed by TMB Development Liquid (Sigma, USA).

### DNA sequence and analysis

All DNA sequencing was done at the URI Genomics and Sequencing Center (University of Rhode Island, Kingston, RI), using an ABI 3170xl Genetic Analyzer unit (Applied Biosystems). Multiple alignment and phylogenic tree were analyzed using the Clustal-W method in DNA-STAR Lasergene7 program.

### Fish infection studies

Various *V. anguillarum* strains were tested for virulence with rainbow trout (*Oncorhynchus mykiss*) by intraperitoneal (IP) injection as described by Mou *et al.*[32]. Briefly, *V. anguillarum* cells grown in LB20 supplemented with appropriate antibiotics for 22 h at 27°C were harvested by centrifugation (9,000 × *g*, 5 min, 4°C), washed twice in NSS, and resuspended in NSS (~2 × 10^9^ cells ml^-1^). Initial cell density was estimated by measurement of optical density at 600 nm. The actual cell density of NSS suspensions was determined by serial dilution and spot plating. All fish were examined prior to the start of each experiment to determine that they were free of disease or injury. Fish were anesthetized with tricaine methanesulfonate (Western Chemical, Ferndale, WA), with 100 mg/L for induction and 52.5 mg/L for maintenance. *V. anguillarum* strains were IP-injected into fish in 100 μl NSS vehicle. Fish that were between 15 and 25 cm long were injected with bacteria diluted with NSS at various doses or NSS only as negative control. Five fish were used for each experimental group. Fish inoculated with different bacterial strains were maintained in separate 10-gallon tanks with constant water flow (200 ml/min) at 19 ± 1°C. The tanks were separated to prevent possible cross-contamination. Death due to vibriosis was determined by the observation of gross clinical signs and confirmed by the recovery and isolation of *V. anguillarum* cells resistant to the appropriate antibiotics from the head kidney of dead fish. The presence of the appropriate strains was tested by PCR analysis. Observations were made for 14 days. All fish used in this research project were obtained from the URI East Farm Aquaculture Center. All fish infection protocols were reviewed and approved by the University of Rhode Island Institutional Animal Care and Use Committee (URI IACUC reference number AN06-008-002; protocols renewed 14 January 2013).

## Competing interests

The authors declare that they have no competing interest.

## Authors’ contributions

LL, XM and DRN designed the study. XM and LL created the strains used in this study. LL and XM performed all the assays. LL, XM and DRN wrote the paper. Formatting of the paper was done by XM and DRN. All authors have read and approved the final version of manuscript.
